# Values and legitimized aggression in healthy and mental disorder groups

**DOI:** 10.1192/j.eurpsy.2026.11535

**Published:** 2026-07-31

**Authors:** T. I. Medvedeva, O. Y. Vorontsova, O. M. Boyko, S. N. Enikolopov, M. I. Oleychik, D. N. Bichevina, V. I. Plakunova

**Affiliations:** 1 clinical psychology, Federal State Budgetary Scientific Institution Mental health scientific center; 2 State Budgetary Healthcare Institution “Moscow City Center for the Rehabilitation of Patients with Spinal Cord Injury and Consequences of Infantile Cerebral Palsy Department of Healthcare”, Moscow, Russian Federation

## Abstract

**Introduction:**

There are areas in society where expressions of aggression are acceptable and used to respond. The expansion of areas of legitimized expression of aggression leads to an increase in manifestations of aggression also in illegitimated areas. The identification of values related to legitimization of aggression can show new ways of prevention of aggressive actions.

**Objectives:**

The aim of the study is to identify the relationship between values and legitimized aggression in mental disorder and in a control groups.

**Methods:**

The study involved 158 people: 46 patients of the endogenous mental disorders department (mean age - 20,8±5,7, male -16%), 112 – control group (male – 23%, mean age 19,8±3,7). The groups did not differ statistically by socio-demographic indicators. Legitimized Aggression Questionnaire based on PLAQ (Hogben), BPAQ-24, SCL-90-R, PVQ-RR were used. Subgroups were compared using analysis of variance and correlation analysis in SPSS.

**Results:**

Comparison of groups showed the following significant differences: in the clinical group below 1) on the parameter legitimization of aggression according to scales «policy» (clinical group 31,395 11,322 healthy group 36,250 13,625, p=0,04), «personal experience» (25,535 11,933; 29,714 11,200; p=0,04), «education» (12,907±5,268; 15,571±6,976; p=0,02); 2) on the parameters сommitment to values «Tradition» (8,652±2,718; 7,170±2,355 p=0,00), «Power Resources» (6,935±2,489; 5,500±2,230; p=0,00) «Conservaton» (22,261±5,725; 19,884±4,234; p=0,00), «Self-Enhancement» (11,891±3,701; 10,188±3,880; 0,01). In the clinical group, clinical symptoms (SCL-90-R) are higher on all scales except SOM. Correlations were identified between the integrated measure of legitimized aggression and the level of psychopathological symptoms HOS, PAR, PSY. The remaining results are presented in table 1.

**Table 1.** Correlations of the integral indicator of legitimized aggression with the indicators of aggression BPAQ-24 and values (PVQ-RR) Table 1. Long description.

	Control group	Clinical group
**BPAQ-24**		
Physical Aggression	,659**	,723**
Anger	,198*	0,288
Hostility	,252**	0,207
**PVQ-RR **		
Conservaton	-0,098	0,031
Openness to change	0,037	-,527**
Self-Enhancement	0,025	-,464**
Self-Transcendence	,339**	,338*

**Conclusions:**

The correlation between the value of «Self-transcendence» and the level of legitimized aggression common to both groups can illustrate the widespread justification of aggressiveness by high ideals. Differences in values associated with the social legitimization of aggression in clinical and healthy groups can be explained by the importance of maintaining stability for people with mental disorders.

**Disclosure of Interest:**

None Declared

## Long descriptions

### Table 1. Long description

The table consists of three columns. The first column lists the indicators, while the second and third columns provide correlation values for the Control group and Clinical group respectively.

Section 1: B P A Q 24 indicators

* Physical Aggression: Control group ,659 double asterisk; Clinical group ,723 double asterisk.

* Anger: Control group ,198 asterisk; Clinical group 0,288.

* Hostility: Control group ,252 double asterisk; Clinical group 0,207.

Section 2: P V Q R R indicators

* Conservation: Control group -0,098; Clinical group 0,031.

* Openness to change: Control group 0,037; Clinical group -,527 double asterisk.

* Self-Enhancement: Control group 0,025; Clinical group -,464 double asterisk.

* Self-Transcendence: Control group ,339 double asterisk; Clinical group ,338 asterisk.

Navigate back to Table 1..

